# The potential drug for treatment in pancreatic adenocarcinoma: a bioinformatical study based on distinct drug databases

**DOI:** 10.1186/s13020-020-00309-x

**Published:** 2020-03-18

**Authors:** Han Liu, Qi Zhou, Wenjuan Wei, Bing Qi, Fen Zeng, Nabuqi Bao, Qian Li, Fangyue Guo, Shilin Xia

**Affiliations:** 1 College of Stomatology, Dalian Medical University, Dalian, China; 2 Institute (College) of Integrative Medicine, Dalian Medical University, Dalian, China; 3 Stem Cell Clinical Research Center, The First Affiliated Hospital of Dalian Medical University, Dalian, Liaoning China; 4 National Joint Engineering Laboratory, The First Affiliated Hospital of Dalian Medical University, Dalian, Liaoning China; 5 Department of General Surgery, The First Affiliated Hospital of Dalian Medical University, Dalian, China; 6 Clinical Laboratory of Integrative Medicine, The First Affiliated Hospital of Dalian Medical University, Dalian, China; 7 Department of Palliative Medicine, Graduate School of Medicine, Juntendo University, Tokyo, Japan

**Keywords:** Chinese traditional medicine database, Pancreatic adenocarcinoma, Differently expressed gene, Potential drug, Molecular docking

## Abstract

**Background:**

The prediction of drug-target interaction from chemical and biological data can advance our search for potential drug, contributing to a therapeutic strategy for pancreatic adenocarcinoma (PAAD). We aim to identify hub genes of PAAD and search for potential drugs from distinct databases. The docking simulation is adopted to validate our findings from computable perspective.

**Methods:**

Differently expressed genes (DEGs) of PAAD were performed based on TCGA. With two Cytoscape plugins of CentiScaPe and MCODE, hub genes were analyzed and visualized by STRING analysis of Protein–protein Interaction (PPI). The hub genes were further selected with significant prognostic values. In addition, we examined the correlation between hub genes and immune infiltration in PAAD. Subsequently, we searched for the hub gene-targeted drugs in Connectivity map (Cmap) and cBioportal, which provided a large body of candidate drugs. The hub gene, which was covered in the above two databases, was estimated in Traditional Chinese Medicine Systems Pharmacology (TCMSP) and Herbal Ingredients’ Targets (HIT) database, which collected natural herbs and related ingredients. After obtaining molecular structures, the potential ingredient from TCMSP was applied for a docking simulation. We finalized a network connectivity of ingredient and its targets.

**Results:**

A total of 2616 DEGs of PAAD were identified, then we further determined and visualized 24 hub genes by a connectivity analysis of PPI. Based on prognostic value, we identified 5 hub genes including AURKA (p = 0.0059), CCNA2 (p = 0.0047), CXCL10 (p = 0.0044), ADAM10 (p = 0.00043), and BUB1 (p = 0.0033). We then estimated tumor immune correlation of these 5 hub genes, because the immune effector process was one major result of GO analysis. Subsequently, we continued to search for candidate drugs from Cmap and cBioportal database. BUB1, not covered in the above two databases, was estimated in TCMSP and HIT databases. Our results revealed that genistein was a potential drug of BUB1. Next, we generated two docking modes to validate drug-target interaction based on their 3D structures. We eventually constructed a network connectivity of BUB1 and its targets.

**Conclusions:**

All 5 hub genes that predicted poor prognosis had their potential drugs, especially our findings showed that genistein was predicted to target BUB1 based on TCMSP and docking simulation. This study provided a reasonable approach to extensively retrieve and initially validate putative therapeutic agents for PAAD. In future, these drug-target results should be investigated with solid data from practical experiments.

## Background

Pancreatic adenocarcinoma (PAAD), which comprises > 85% of all pancreatic cancer cases [1], remains refractory among solid cancers, and therefore is the focus of the pancreatic cancer studies. The survival rate of PAAD remains bleak with a five-year overall survival of 9% [2]. Currently, the economic burden of PAAD stays high among the major malignancies [3, 4]. The chemical entities and natural reagents are being developed for decades, and most of them may be redesigned and examined so many times. Even though some of these drugs are approved by the Food and Drug Administration (FDA), majority are halted either in laboratory or in clinical trials after time consume and efforts cost. As a result, highly effective methods of initial identification for candidate drug become imperative for advancing targeted drug for cancer therapy. With technological advance and tool innovation, many resources and databases have empowered in depth studies on the mechanisms accounting for cancer treatment [5, 6]. The drug-target investigation has been ongoing to discover novel therapeutics for pancreatic cancer [7, 8]. However, whether these findings will translate to the clinical setting is not known. Given the complementary resources of different drug databases that constitute an important aspect of drug research, we hypothesized that multiple databases provide candidate drugs, especially for oncogene that is not recorded or predicted in any separate database.

Although drug databases are built to have a repertoire of pharmaceutical ingredients and therapeutic agents, it is difficult to cover all oncogenes [9]. It was known that oncogene is associated with the risk of tumorigenesis and the risk increases with aberrant activation of multiple oncogenes [10, 11]. Therefore, a subset of high-risk oncogenes should be targeted by candidate drugs from a computational screening and a solid validation, which enable substantial candidate drugs to display advantages in meeting the demands of therapeutic agents in PAAD treatment.

In the present study, we initiated a screening of differently expressed genes (DEGs) using GEPIA, which is based on the Cancer Genome Atlas (TCGA). According to an analysis of Protein–protein Interaction (PPI), we administrated a network connectivity of DEGs in order to determine hub genes. Subsequently, we examined hub genes for Gene Ontology (GO) analysis and pathway enrichment, as well as prognostic value in PAAD. The immune correlation analysis was additionally performed for hub genes that predicted poor prognosis. Next, the Connectivity map (Cmap) and cBioportal database were used to retrieve candidate drugs for the above hub genes. Traditional Chinese Medicine Systems Pharmacology Database (TCMSP) and Herbal Ingredients’ Targets (HIT) were applied to show related ingredients for the hub gene that was covered neither in Cmap nor cBioportal. Based on RCSB PDB and PubChem, we obtained structures of candidate drug and its target in order to simulate molecular docking using a web tool of Swissdock and a software of USCF Chimera. At final, we provided an analysis of drug-target network as a landscape to give new insights on a prospective application of potential drugs.

## Methods

### Identification of differently expressed genes in PAAD

We carried out DEGs collection of PAAD from GEPIA (http://gepia.cancer-pku.cn/index.html), which is an online server for analyzing RNA sequencing expression data of tumors and normal samples from TCGA [6]. In our study, GEPIA was applied to identify DEGs between PAAD and pancreas tissue. The genes with higher |log_2_fold-change (FC)| values and lower q values than a pre-set threshold was examined and considered as DEGs. We set |log_2_FC| > 2 and q < 0.01 to identify DEGs in PAAD. The GEPIA was also used to generate an expression difference and prognostic value of differently expressed genes.

### Identification and analysis of hub genes

In an attempt to determine which genes may serve as key roles among DEGs, we constructed a landscape of PPI in order to analyze complex network connectivity and identify hub genes in PAAD. We exhibited a network connectivity of DEGs using STRING (http://string-db.org) (version 11.0). The STRING is a web-based tool that provides insights into protein–protein interaction and that reveals a stable steady-state distribution of gene expression [12, 13], which may predict the tumorigenic mechanism of PAAD in this study. The score = 0.900 was recognized as statistically significant. Based on the co-expression analysis of STRING, we showed that hub genes were observed to be correlated in expression, across a large number of experiments.

After initial analysis in STRING website, we continued to screen and visualize network using Cytoscape software (version 3.7.2). Cytoscape, a bioinformatics software with multiple open source modes and plugins, enables a visualization of protein–protein interaction and a cluster screening of complicated molecular network [14]. We prioritized functional assignment in DEGs network using two plugins of Cytoscape including CentiScaPe (version, 3.6.0; http://apps.cytoscape.org/apps/centiscape) and Molecular Complex Detection (MCODE) (version 1.5.1) [15]. The CentiScaPe, designed by Center for Biomedical Computing in University of Verona, describes a network topology with computing specific centrality parameters. All parameters in CentiScaPe were selected in the present study, such as Degree and Betweenness. The MCODE, designed by Bader Lab in University of Toronto, provides a cluster analysis in a protein–protein interaction network. The configuration of MCODE was as follows: Degree Cutoff = 2, Node score Cutoff = 0.2, K-Core = 2, Max. Depth = 100.

### GO analysis and pathway enrichment of hub genes

Gene Ontology consisted of biological process, molecular function, and cellular component [16]. According to the identification of hub genes, we achieved GO enrichment to collect functional annotation hub genes using STRING. In our study, GSCALite (http://bioinfo.life.hust.edu.cn/web/GSCALite/), a web analysis platform based on TCGA, offered the enriched pathway of DEGs between pathway activity groups with activity/inhibition/non-significant effect [17].

### Tumor immune estimation of hub genes

Among biological processes, immune effector process was most significantly involved by hub genes. We further explored the immune infiltrates correlation of 5 hub genes with significant prognostic value in Tumor IMmune Estimation Resource (TIMER) (https://cistrome.shinyapps.io/timer/). Based on TCGA, TIMER was developed by Shirley Liu’s Lab in Dana-Farber Cancer Institute and Jun Liu’s Lab in Department of Statistics at Harvard University. TIMER is an online server for systematical analysis of immune infiltrates across diverse cancer types [18]. We estimated 5 hub genes with six immune infiltrates, including B cells, CD4+ T cells, CD8+ T cells, neutrophils, macrophages and dendritic cells. This study revealed the correlation between 5 hub genes and immune infiltration in PAAD.

### Potential drug for hub genes with significant prognostic value

We distinguished the hub gene with p < 0.05, which was significantly associated with survival of PAAD patient. The Connectivity map database (https://www.broadinstitute.org/connectivity-map-cmap), designed by Broad Institute of MIT and Harvard, uses gene‑expression signatures to predict small molecular compounds for a specific disease [19, 20]. Here we uploaded all hub genes to the “query” of Cmap, then obtained small molecule drugs as candidate agents/drugs. Then, these hub genes were submitted in “query gene” of PAAD in cBioPortal (http://www.cbioportal.org/). The cBioPortal, an online server based on TCGA, was used to construct a drug-target network [21]. As a result, we obtained potential drugs for hub genes with the concurrent use of both Cmap and cBioPortal.

### The candidate drug from TCMSP and HIT

Except for both Cmap and cBioPortal, we selected alternate databases to guide our research in case of hub gene without targeted drug. The TCMSP database was applied to search for candidate drug when hub gene was covered as target neither in Cmap nor cBioPortal. TCMSP (version 2.3) is a unique systems pharmacology platform of Chinese herbal medicines that captures the relationships between drugs, targets and diseases (http://tcmspw.com/index.php). Under TCMSP mode, we input hub gene in “Target name” and retrieved related ingredients as targeted drug. The result from TCMSP was validated in HIT database. We input the candidate drug from TCMSP into HIT database in order to verified the prediction of potential ingredient. The HIT database (http://lifecenter.biosino.org/hit/welcome.html) is a comprehensive and fully curated database for Herbal Ingredients’ targets [22]. Derived from more than 3250 literatures, HIT currently contains 5208 entries about 1301 known protein targets.

### The molecular docking simulation on BUB1 and genistein

To further understanding the docking mode of drug-target, we obtained 3D structures of BUB1 from RCSB PDB (http://www.rcsb.org) and 3D structures of genistein from PubChem (https://pubchem.ncbi.nlm.nih.gov). Then we simulated a molecular docking using Swissdock (http://www.swissdock.ch/docking). This web server, designed by Swiss Institute of Bioinformatics, is performed to predict the molecular interactions [23]. We uploaded 3D structure of BUB1 as “Target selection” and that of genistein as “Ligand selection” prior to a result report.

The USCF Chimera software (version 1.15), a molecular modeling system, was used to calculate possible binding modes and present an interactive visualization of data from Swissdock. The best two docking simulations was sorted by FullFitness, as well as Energy by which a specific ligand acts on a complex protein.

### The network of genistein and its targeted proteins based on STITCH

For the exploration of the association between BUB1 and genistein, we established an interaction network using STITCH database (version 5.0) (http://stitch.embl.de). The STITCH is a database for predicted interactions between chemicals and proteins [24]. The interaction network was finally visualized with Cytoscape (version 3.7.2) to elucidate the association between genistein and its targets, as well as BUB1.

### Statistical analysis

We applied one‑way ANOVA method and Tukey’s test to compare gene expression between PAAD and non‑PAAD adjacent tissues. DEG expression pattern was determined with p < 0.05. We carried out Log-rank test, as known as the Mantel–Cox test to analyze overall survival. The cox proportional hazard ratio and the 95% confidence interval information are measured in overall survival analysis. Gene with p < 0.01 was considered to have a significant prognostic value. The purity-corrected partial Spearman’s correlation was performed in tumor immune estimation. p < 0.05 was considered statistically significant in tumor immune estimation.

## Results

### Identification of differently expressed genes in PAAD

**Fig. 1:**
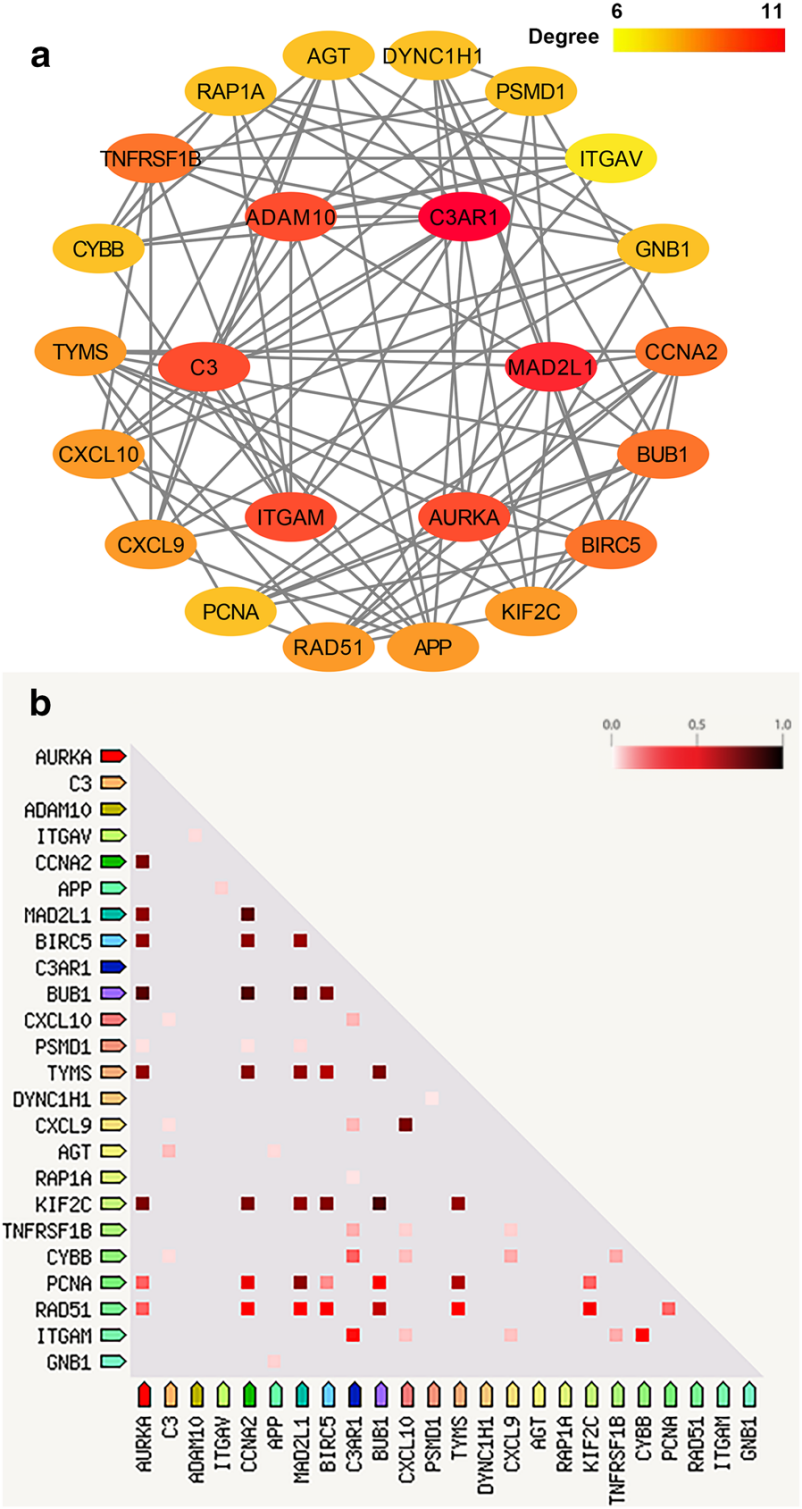
The network of hub genes with degree constructed by Cytoscape. **a** Two circles represented the protein–protein interaction from degrees 6 to 11. The hub genes with high degree in inner circle, and hub genes with low degree in outer circle. **b** The co-expression of hub genes. The deeper color represented the co-expression between two genes

**Fig. 2:**
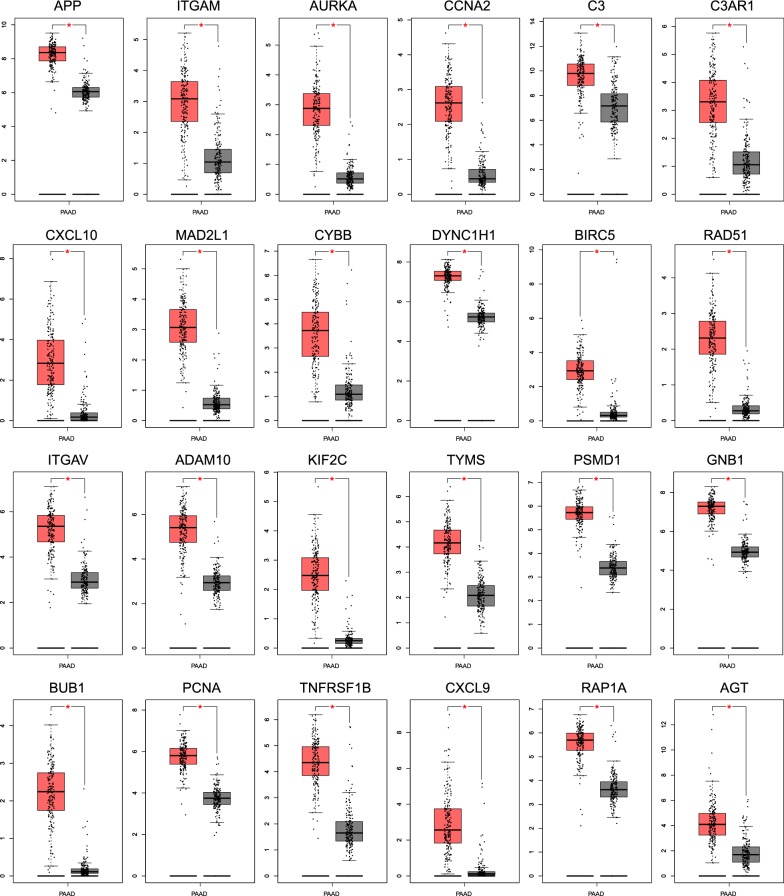
The expression difference of hub genes in pancreatic adenocarcinoma. *p < 0.05

From GEPIA database, we identified 2616 DEGs in PAAD, including 2458 upregulated and 158 downregulated genes located on chromosomes (Additional file 1: Fig. S1). We used STRING to investigate a network connectivity of all DEGs. Next, we continued to prioritize connective assignment with the concurrent use of two Cytoscape plugins. Our results demonstrated that 24 upregulated hub genes had a significant network connectivity (Fig. 1a), which revealed that these highly connected proteins had a closer response to biological stimuli in PAAD. An additional result of co-expression also gave an evidence that highly connected proteins were functionally associated in homo sapiens (Fig. 1b). We then examined the expression pattern of 24 hub genes to validate that these hub genes were significantly upregulated (Fig. 2). These results indicated that aberrant activation of 24 hub genes played an important role on tumorigenicity in pancreatic adenocarcinoma.

### GO analysis and pathway enrichment of hub genes in PAAD

To further understanding of biological function of 24 hub genes, we described GO analysis in three categories, including biological process, molecular function, and cellular component (Table 1). From the biological process, 13 DEGs involved in immune effector process with minimum false discovery rate, suggesting that the most connected proteins played an important role on an immune response process of PAAD. Form molecular function, a total of 22 hub genes showed protein binding function, except BUB1 and C3AR1. In cellular component, most of DEGs were associated with granule of cell.

**Table 1:** GO terms analysis for each GO category

Categories	Term ID	Term description	False discovery rate	Matching proteins in your network (labels)
Biological process	GO:0002252	Immune effector process	2.01E-08	ADAM10,APP,C3,C3AR1,CXCL10,CXCL9,CYBB,DYNC1H1,ITGAM,ITGAV,PSMD1,RAP1A,TNFRSF1B
	GO:0001775	Cell activation	2.70E-08	ADAM10,APP,C3,C3AR1,CXCL10,CYBB,DYNC1H1,GNB1,ITGAM,ITGAV,PSMD1,RAP1A,TNFRSF1B
	GO:0002274	Myeloid leukocyte activation	2.70E-08	ADAM10,APP,C3,C3AR1,CYBB,DYNC1H1,ITGAM,ITGAV,PSMD1,RAP1A,TNFRSF1B
	GO:0002263	Cell activation involved in immune response	3.03E-08	ADAM10,APP,C3,C3AR1,CYBB,DYNC1H1,ITGAM,ITGAV,PSMD1,RAP1A,TNFRSF1B
	GO:0032940	Secretion by cell	5.54E-08	ADAM10,AGT,APP,C3,C3AR1,CYBB,DYNC1H1,ITGAM,ITGAV,PSMD1,RAP1A,TNFRSF1B
Molecular function	GO:0005515	Protein binding	1.40E-06	ADAM10,AGT,APP,AURKA,BIRC5,C3,CCNA2,CXCL10,CXCL9,CYBB,DYNC1H1,GNB1,ITGAM,ITGAV,KIF2C,MAD2L1,PCNA,PSMD1,RAD51,RAP1A,TNFRSF1B,TYMS
	GO:0001664	G protein-coupled receptor binding	0.00011	AGT,APP,C3,CXCL10,CXCL9,GNB1
	GO:0019899	enzyme binding	0.00026	ADAM10,APP,AURKA,BIRC5,CCNA2,GNB1,ITGAV,PCNA,PSMD1,RAD51,RAP1A,TNFRSF1B
	GO:0048248	CXCR3 chemokine receptor binding	0.0019	CXCL10,CXCL9
	GO:0004866	Endopeptidase inhibitor activity	0.0023	AGT,APP,BIRC5,C3
Cellular component	GO:0035579	Specific granule membrane	5.81E -09	ADAM10,C3AR1,CYBB,ITGAM,ITGAV,RAP1A,TNFRSF1B
	GO:0030141	Secretory granule	1.30E-07	ADAM10,APP,C3,C3AR1,CYBB,DYNC1H1,ITGAM,ITGAV,PSMD1,RAP1A,TNFRSF1B
	GO:0000793	Condensed chromosome	1.12E-05	AURKA,BIRC5,BUB1,KIF2C,MAD2L1,RAD51
	GO:0000779	Condensed chromosome, centromeric region	1.46E-05	AURKA,BIRC5,BUB1,KIF2C,MAD2L1
	GO:0044433	Cytoplasmic vesicle part	1.46E-05	ADAM10,APP,C3,C3AR1,CYBB,DYNC1H1,ITGAM,ITGAV,PSMD1,RAP1A,TNFRSF1B

**Fig. 3:**
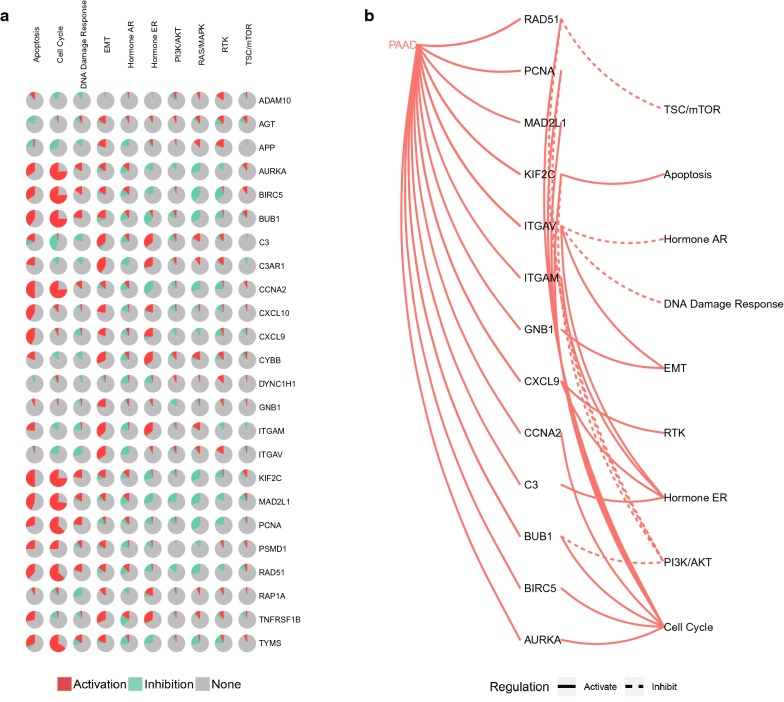
The pathway enrichment analysis of hub genes. **a** The pathway analysis of hub genes represented by the pie chart. **b** The regulation of hub genes in pathways, including activation and inhibition

We investigated the pathway enrichment of 24 hub genes, since oncogenic protein generally involved multiple pathways to drive the development of PAAD. We found that 24 hub genes played a varying role on distinct pathways (Fig. 3). It was apparently observed that cell cycle was the most active pathway involved by AURKA, BIRC5, BUB1, CCNA2, KIF2C, MAD2L1, PCNA, RAD51, and TYMS. Interestingly, almost the same subset of DEGs remarkedly inhibited RAS/MAPK pathway. The results in this chapter exhibited the biological function and signal pathway of 24 hub genes in PAAD (Table 1).

### Prognostic value of hub genes analysis

**Fig. 4:**
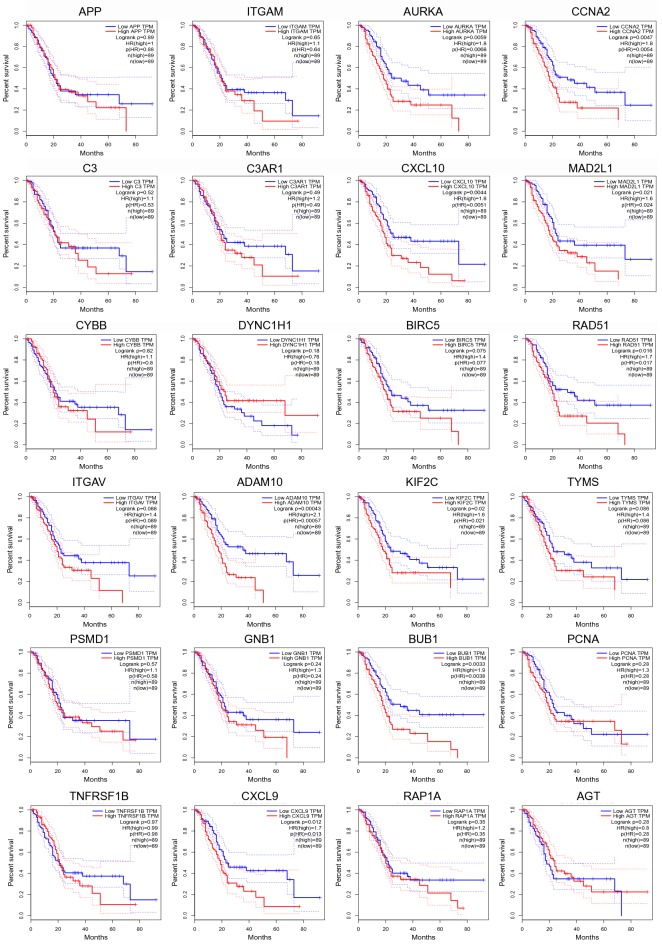
Prognostic value of hub genes in pancreatic adenocarcinoma

Regarding that hub genes highly involved in tumorigenesis of PAAD, we carried out overall survival analysis in order to determine prognostic value of hub gene (Fig. 4). Among 24 hub genes, 5 hub genes performed significant prognostic value (p < 0.01) in PAAD, including AURKA (p = 0.0059), CCNA2 (p = 0.0047), CXCL10 (p = 0.0044), ADAM10 (p = 0.00043), and BUB1 (p = 0.0033).

**Fig. 5:**
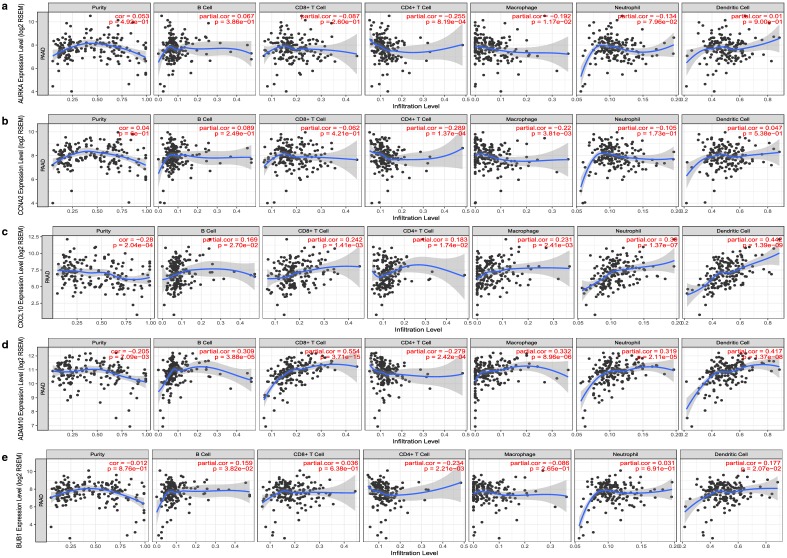
The immune infiltrates estimation of hub genes with significant prognostic value, including **a** AURKA, **b** CCNA2, **c** CXCL10, **d** ADAM10, and **e** BUB1

Given that immune effector process was the most significant biological process in GO analysis, we evaluated the tumor immune estimation of 5 hub genes that had significant prognostic value (Fig. 5). We observed that CXCL10 and ADAM10 had negative associations with tumor purity. During immune infiltration of PAAD, the most involved immunocyte was CD4+ T cell, which was negatively associated with AURKA, CCNA2, ADAM10 and BUB1. Among 5 hub genes, AURKA and BUB1 had almost no participation in immune response of PAAD. Together these results provided the prognostic value of 24 hub genes and the correlation between six immune infiltrates and hub genes.

### Identification of candidate drug for PAAD treatment

In total, 5 genes among 24 hub genes were selected by significant prognostic value (p < 0.01). The next question was to identify candidate drugs that were available to target hub genes. From Cmap and cBioportal, we aimed to search for candidate drug (after removing duplicates) that targeted these 5 hub genes. In Cmap database, we found 16 drugs targeted for these 5 hub genes (Additional file 2: Table S1). In a landscape of network from cBioportal, we observed that there were a total of 86 drugs for all 24 hub genes, and all drugs comprised 14 drugs approved by FDA and 72 drugs not approved by FDA (Additional file 3: Fig. S2). For the 5 hub genes, all 49 drugs were not approved by FDA.

Of interest, BUB1 was covered neither in Cmap nor cBioportal. Besides, a drug for BUB1 is not known even in DrugBank database. Thus, we turned to TCMSP and HIT database to find whether there was any ingredient from two databases of traditional Chinese medicine. In TCMSP, genistein, a soy-derived isoflavone and phytoestrogen, was finally exhibited as a potential chemical molecular that targeted BUB1. In HIT, genistein was validated to target BUB1, the target ID was T0508 (http://lifecenter.biosino.org/hit/search.jsp). In the next section of this study, we attempted to simulate an interaction mode between BUB1 and genistein.

### Molecular docking simulation and network connective analysis

**Fig. 6:**
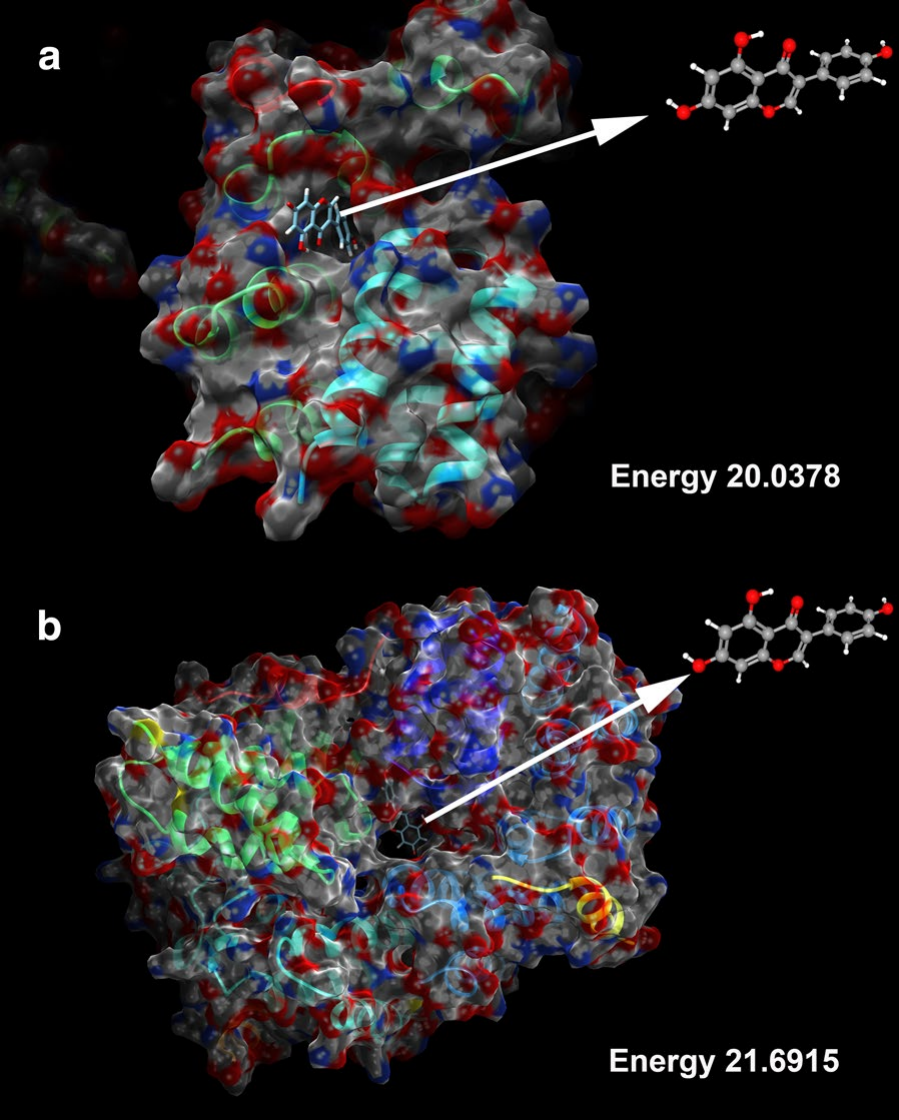
Molecular docking simulation for genistein and BUB1. **a** One simulation mode with Energy 21.6915. **b** The other mode with Energy 20.0378

For the purpose of docking, we obtained a structure of Bub1 kinase domain (PDB code: 4QPM) and 3D structures of genistein (PubChem CID: 5280961). The Swissdock generated a series of docking modes with Energy score from 16.6623 to 34.6584. The lower the energy stood for the more favorable docking mode. Consequently, two modes were emerged as putative docking modes for genistein and its target BUB1 (Fig. 6).

**Fig. 7:**
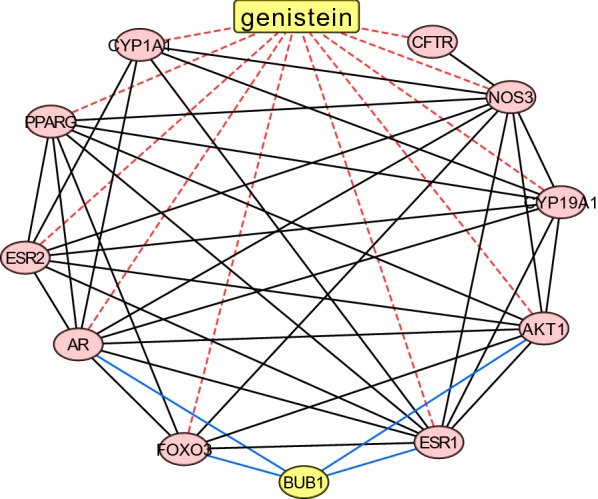
The network between BUB1 and genistein’s targets. Blue edge represented the interaction between BUB1 and genistein’s targets. Red edge represented the correlation between genistein and its targets based on STITCH database

We then explored a connectiyity underlying the network between genistein’s target proteins and BUB1. Our results demonstrated there were 10 proteins that targeted by genistein (Fig. 7), including CYP1A1, PPARG, ESR2, AR, FOXO3, ESR1, AKT1, CYP19A1, NOS3, and CFTR. Among these proteins, BUB1 connected with 4 proteins: FOXO3, ESR1, AR, and AKT1. These results indicated that genistein was not only an ingredient for BUB1, but also a potential ligand that regulated alternative proteins, which had a molecular interaction with BUB1.

## Discussion

In the present study, we identified DEGs of PAAD by collecting data from TCGA and carried out a bioinformatics approach to search for hub gene-targeted drugs from distinct drug databases. Among Cmap, cBioportal and even DrugBank, however, we had no candidate drug for BUB1, which presented a prognostic risk factor in PAAD. We finally found evidence from TCMSP, a traditional medicine database for predicting drug-target interaction based on Random Forest (RF) and Support Vector Machine (SVM) [25]. The practicality of this new result was then tested on web of Swissdock, which exhibited two docking modes between BUB1 and genistein. Based on the prediction analysis and docking result, our findings showed the available evidence of our hypothesis that candidate drugs from distinct databases were predicted and simulated for oncogenes in order to provide more optional compatibility of drugs, contributing to some insight into therapeutic strategy.

Pancreatic adenocarcinoma is highly aggressive, and the prognosis remains dismal [26, 27]. Malignant growth of cancer depends on cell proliferation and division. BUB1, known as a mitotic checkpoint serine/threonine-protein kinase, is essential for spindle-assembly checkpoint signaling and for correct chromosome alignment [28]. It obviously indicated that BUB1 may enable its use for a promoter of cell proliferation. The oncogenic role of BUB1 accords with its prognostic impact that we had achieved by a bioinformatics method. With BUB1, a subset of proteins involves in the regulation of checkpoint and a complex process of mitosis, in order to render cancer cell to keep proliferative capacity [29]. In our study, we performed a drug-target network to explore that BUB1 were connected with AR, ESR1, AKT1, and FOXO3 under a scenario of genistein-target network. AR and ESR1 involved in the regulation of gene expression and affect cellular proliferation [30, 31]. AKT1 mediated many processes including metabolism, proliferation, cell survival, growth and angiogenesis [32]. FOXO3 triggers apoptosis in the absence of survival factors, including neuronal cell death upon oxidative stress [33]. All above proteins play an important role on tumorigenesis and development of PAAD. Actually, it’s difficult to realize a medical combination for various targets. The fact that multiple proteins drive malignant proliferation of cancer cell pleas for a better understanding of the comprehensive therapeutics by which medical scientists construct and utilize an approach for drug retrieval and combination.

It is generally known that time consume and economic costs are enormous in drug search and development. The rational drug selection and combination for cancer therapy may emerged with the concurrence use of different drug resources, especially the drug databases that have harnessed different algorithm for selecting ingredients and herbs, or that have collected more variety of drug with historical and regional characteristics. In this study, we first determined hub genes from DEGs based on TCGA database. Second, we preferred hub genes in dependent on their prognostic values and searched for drugs from Cmap, cBioportal, and DrugBank. Then, we turned to TCMSP database in a final attempt to retrieve related ingredients for hub gene, since hub gene was not covered in the above three databases. Third, we validated the potential ingredient from TCMSP using HIT database, another resource of Herbal Ingredients’ targets. We eventually obtained genistein in a TCMSP database. Besides, we had retrieved candidate ingredients in Traditional Chinese Medicines Integrated Database (TCMID) (http://119.3.41.228:8000/tcmid/), yet there was no alternative ingredient. Genistein can extracted from a number of Chinese herbs, such as *Iridis Tectori Rhizoma*, *Sojae Semen Praeparatum* and *Eucommiae Cortex*. The utility of these herbs is recorded in traditional medical books in China, which is consistent with contemporary research [34]. Previous studies provided some evidence that genistein was recognized to inhibit the uncontrolled cell growth of cancer [34, 35]. Extensive research has shown that moderate doses of genistein have inhibitory effects on cancers of the prostate [36, 37], cervix [38], brain [39], breast [40, 41] and colon [42]. Therefore, our findings bring some insights into the application of potential drug like genistein for PAAD treatment.

In this study, the results showed that immune effector process was the most prominent one in biological process of GO analysis. Our findings may support an evidence that immunotherapy has been proving itself as an effective therapeutic strategy. In 2013, immunotherapy was deemed as “Breakthrough of the Year” in Science journal. In 2015, the FDA approved PD-1/PD-L1 immunotherapies to treat the most common forms of advanced lung and kidney cancer. The American Society of Clinical Oncology (ASCO) announced immunotherapy as the top cancer advance in two consecutive years from 2016 to 2017. The immunotherapy, such as chimeric antigen receptor therapy, is extensively studied and applied in recent years [43, 44]. Immunotherapy plays an expanding role on cancer treatment. However, the development curve of immunotherapy is fluctuating. Some unnerving side effects are observed. It is exemplified that the uncontrolled release of cytokines bring inflammation during CAR T cell therapy [45, 46], which can also induce neurotoxicity with symptoms like dyslexia, and dyskinesia [47, 48]. The impediments of immunotherapy give a reason that chemotherapeutic agents are available to improve the stability of immunotherapy. On the one hand, we found that only two hub genes (CXCL10 and ADAM10) had negative associations with tumor purity. However, these two hub genes had extensively positive correlation with four immune infiltrates, including CD8+ T cell, macrophage, neutrophil, and dendritic cell. This finding reflected that there might be a subset of hub genes which played their roles on a wide participation of immune response. On the other hand, an additional observation showed that four hub genes had was negatively associated with CD4+ T cell, while they were not related with other immune infiltrates. It seemed that there were a subset of hub genes which had an effect on single category of immune infiltrates. In future, integrative therapy between targeted chemotherapy and immunotherapy may become an approach to PAAD treatment.

In this study, some limitation remains to be answered. First, the docking simulation was to predict two interaction modes of BUB1 and genistein on computer screen, rather than an observation on experimental table, so it needs to be validated whether our docking result was applicable to actual interaction. Second, the effect of potential drugs from Cmap and cBioportal for PAAD treatment should also be further investigated using practical evidence. Based on more than ten online tools and databases, we aimed to explore the potential drug for hub gene in PAAD. Third, the hub genes verified from STRING and Cytoscape should be further validated in solid experiments to observe their specific roles in PAAD. In future, our in silico analysis should be detected and verified from experimental data in extensive experiments.

## Conclusion

In summary, the evidence from this study suggested that the drug which targeted for hub gene might be exploited as a therapeutic drug for PAAD treatment. We searched for DEGs from a resource of TCGA database. Based on identification from analysis of network connectivity, hub genes had been investigated by GO analysis and pathway enrichment. Subsequently, potential drugs were searched from distinct databases. Furthermore, we provided that genistein may have some target sites on BUB1 to support the hypothesis that a novel drug-target correlation served for comprehensive therapy may be emerged by a prediction from drug database and a simulation by molecular docking. The investigation should be further carried out with practical data from a series of concrete experiments.

## Supplementary information

**Additional file 1: Fig.** **1.** All 2616 differently expressed genes of PAAD located on chromosomes. Red represented over-expressed genes in PAAD. Green represented under-expressed genes.

**Additional file 2: Table S1.** Prediction drug for hub genes based on Cmap database.

**Additional file 3: Fig.** **2.** Interaction network analysis of 24 hub genes. Nodes with bold black outline represented hub genes. Yellow hexagon represented the drug approved by FDA, and White hexagon represented the drug not approved by FDA.

## Data Availability

The datasets generated and/or analyzed during the current study are available as followed: GEPIA (http://gepia.cancer-pku.cn/index.html). STRING (http://string-db.org). CentiScaPe (version, 3.6.0; http://apps.cytoscape.org/apps/centiscape). GSCALite (http://bioinfo.life.hust.edu.cn/web/GSCALite/) TIMER (https://cistrome.shinyapps.io/timer/). Cmap database (https://www.broadinstitute.org/connectivity-map-cmap). cBioPortal (http://www.cbioportal.org/). TCMSP (http://tcmspw.com/index.php). HIT database (http://lifecenter.biosino.org/hit/welcome.html). PDB (http://www.rcsb.org) PubChem (https://pubchem.ncbi.nlm.nih.gov). Swissdock (http://www.swissdock.ch/docking). STITCH database (version 5.0) (http://stitch.embl.de). TCMID (http://119.3.41.228:8000/tcmid/).

## Abbreviations

PAAD: Pancreatic adenocarcinoma
DEGs: Differently expressed genes
PPI: Protein-protein interaction
Cmap: Connectivity map
FDA: The food and drug administration
TCGA: The cancer genome atlas
GO: Gene ontology
TCMSP: Traditional Chinese medicine systems pharmacology database
HIT: Herbal ingredients’ targets
FC: Fold-change
MCODE: Molecular complex detection
TIMER: Tumor immune estimation resource
APP: Amyloid-beta A4 protein
ITGAM: Integrin alpha-M
AURKA: Aurora kinase A
CCNA2: Cyclin-A2
C3: Complement C3
C3AR1: C3a anaphylatoxin chemotactic receptor
CXCL10: C-X-C motif chemokine 10
MAD2L1: Mitotic spindle assembly checkpoint protein MAD2A
CYBB: Cytochrome b-245 heavy chain
DYNC1H1: Cytoplasmic dynein 1 heavy chain 1
BIRC5: Baculoviral IAP repeat containing 5
RAD51: DNA repair protein RAD51 homolog 1
ITGAV: Integrin alpha-V
ADAM10: Disintegrin and metalloproteinase domain-containing protein 10
KIF2C: Kinesin-like protein KIF2C
TYMS: Thymidylate synthase
PSMD1: 26S proteasome non-ATPase regulatory subunit 1
GNB1: Guanine nucleotide-binding protein G(I)/G(S)/G(T) subunit beta-1
BUB1: Mitotic checkpoint serine/threonine-protein kinase
PCNA: Proliferating cell nuclear antigen
TNFRSF1B: Tumor necrosis factor receptor superfamily member 1B
CXCL9: C-X-C motif chemokine 9
RAP1A: Ras-related protein rap-1A
AGT: Angiotensinogen
