# Supplementary figures and images for: The potential drug for treatment in pancreatic adenocarcinoma: a bioinformatical study based on distinct drug databases
Source: Chin Med. 2020 Mar 18;15:26. doi: 10.1186/s13020-020-00309-x (PMC7079489; doi:10.1186/s13020-020-00309-x)

# The Differentially Expressed Genes On Chromosomes

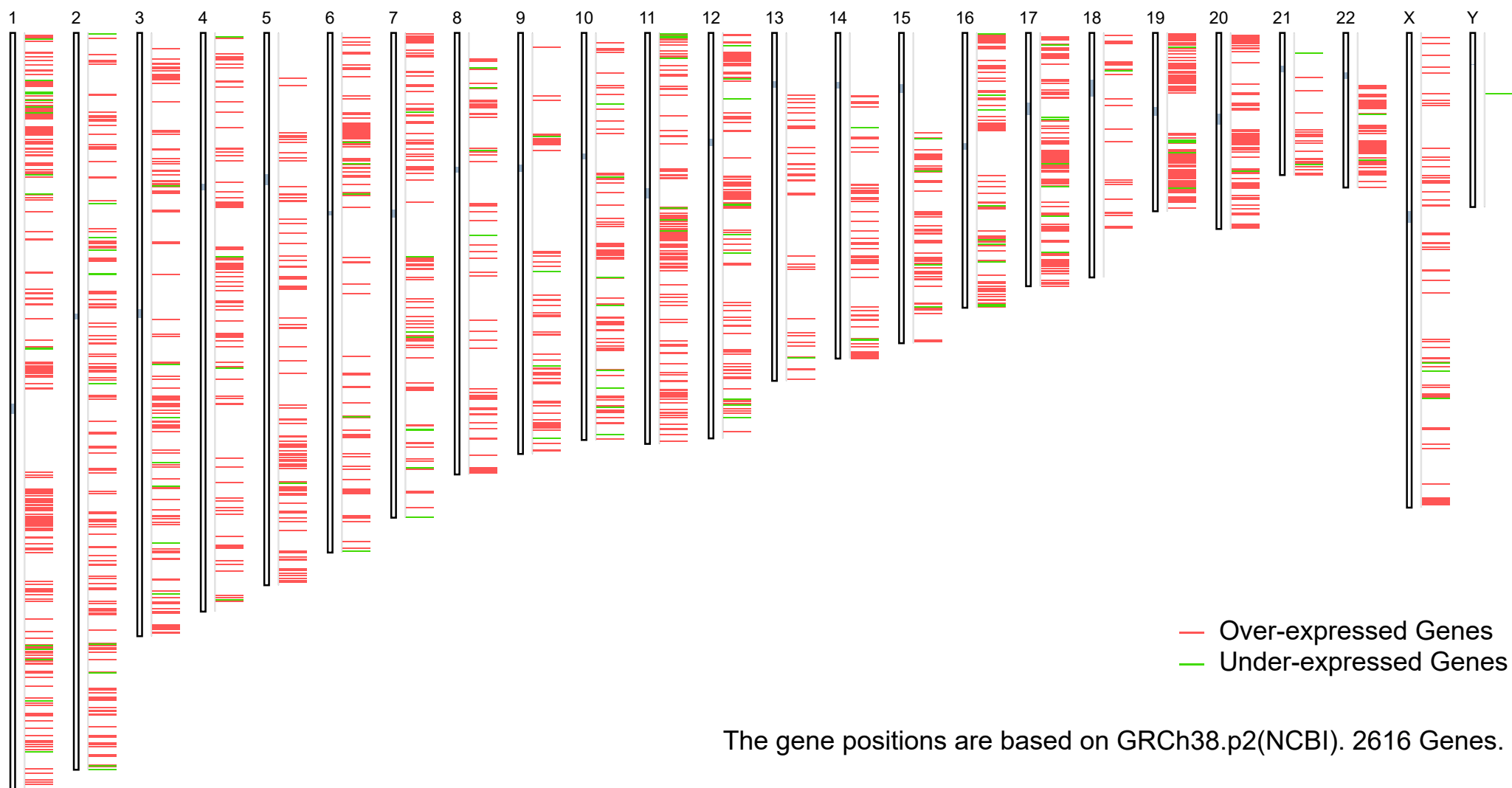

Supplement: Supplementary file 1 — Additional file 1: Fig. 1. All 2616 differently expressed genes of PAAD located on chromosomes. Red represented over-expressed genes in PAAD. Green represented under-expressed genes. [file 13020_2020_309_MOESM1_ESM.pdf]

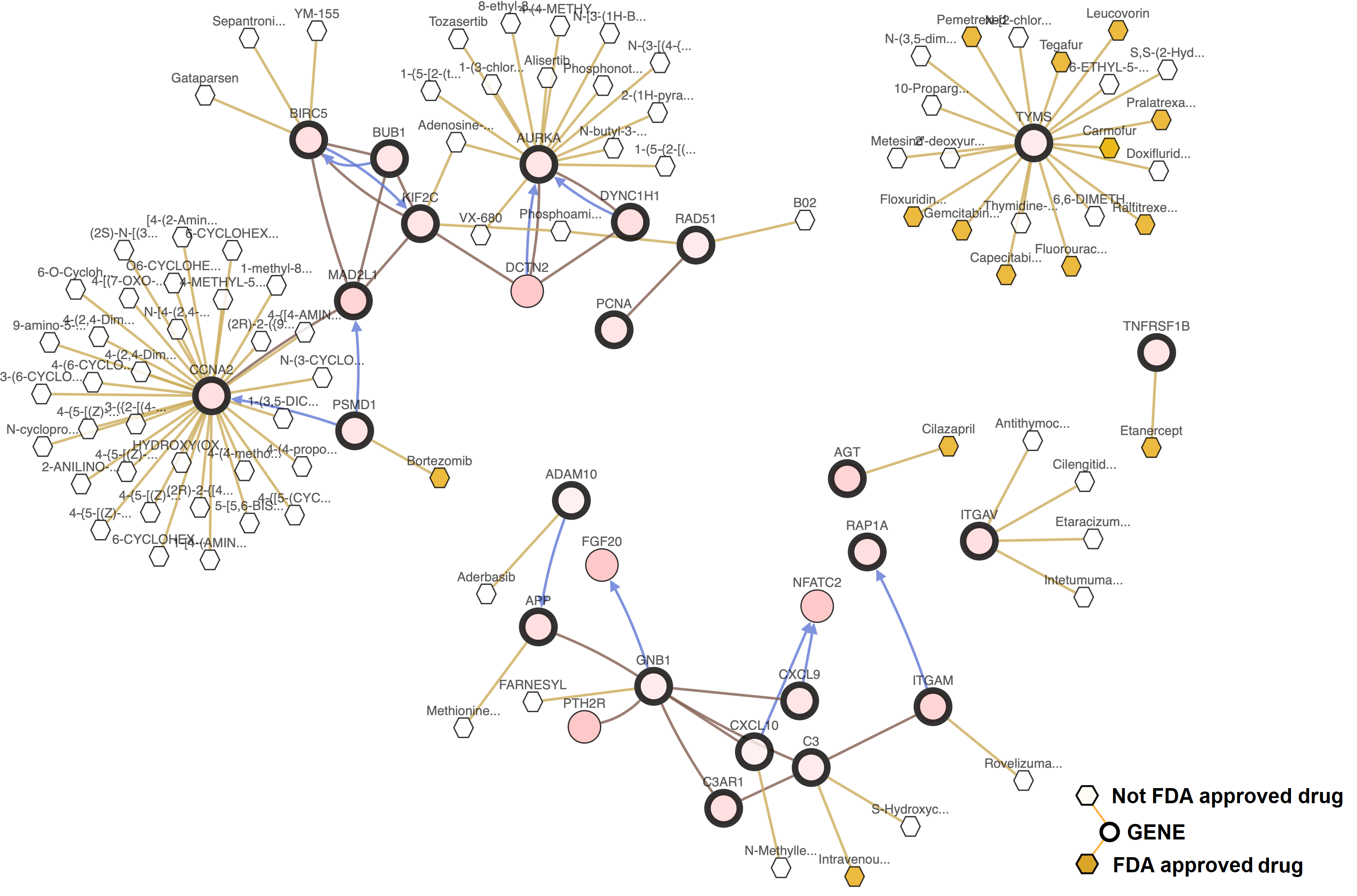

Supplement: Supplementary file 3 — Additional file 3: Fig. 2. Interaction network analysis of 24 hub genes. Nodes with bold black outline represented hub genes. Yellow hexagon represented the drug approved by FDA, and White hexagon represented the drug not approved by FDA. [file 13020_2020_309_MOESM3_ESM.tif]
