# Supplementary material for: The potential drug for treatment in pancreatic adenocarcinoma: a bioinformatical study based on distinct drug databases
Source: Chin Med. 2020 Mar 18;15:26. doi: 10.1186/s13020-020-00309-x (PMC7079489; doi:10.1186/s13020-020-00309-x)
Supplement: Supplementary file 2 — Additional file 2: Table S1. Prediction drug for hub genes based on Cmap database. [file 13020_2020_309_MOESM2_ESM.docx]

Table 1S Prediction drug for hub genes based on Cmap database.

| **Rank** | **Score** | **Type** | **ID** | **Name** | **Description** | **Target** | **MOA** |
| --- | --- | --- | --- | --- | --- | --- | --- |
| 1340 | 55.76 | cp | BRD-K71512533 | SNS-314 | Aurora kinase inhibitor | AURKA, AURKB, AURKC | Aurora kinase inhibitor |
| 41 | 99.68 | cp | BRD-K83963101 | MLN-8054 | Aurora kinase inhibitor | AURKA | Aurora kinase inhibitor, Mitotic inhibitor, Protein kinase inhibitor |
| 70 | 99.4 | cp | BRD-K53665955 | MK-5108 | Aurora kinase inhibitor | AURKA, AURKB, AURKC | Aurora kinase inhibitor |
| 109 | 98.94 | cp | BRD-K59369769 | tozasertib | Aurora kinase inhibitor | AURKA, AURKB, ABL1, AURKC, BCR, FLT3, JAK2, DDR2, LCK | Aurora kinase inhibitor, BCR-ABL kinase inhibitor, FLT3 inhibitor, JAK inhibitor |
| 1371 | 54.38 | cp | BRD-K72703948 | ZM-447439 | Aurora kinase inhibitor | AURKA, AURKB | Aurora kinase inhibitor |
| 2523 | 15.07 | cp | BRD-K91696562 | orantinib | FGFR inhibitor | PDGFRB, AURKA, AURKB, KDR, EGFR, FGFR1, FGFR2, PDGFRA, TBK1 | FGFR inhibitor, VEGFR inhibitor, PDGFR receptor inhibitor |
| 5197 | -46.46 | cp | BRD-K75295174 | alisertib | Aurora kinase inhibitor | AURKA | Aurora kinase inhibitor |
| 5990 | -64.98 | cp | BRD-K53561341 | KIN001-220 | Aurora kinase inhibitor | AURKA | Aurora kinase inhibitor |
| 8014 | -98.63 | cp | BRD-K68488863 | ENMD-2076 | FLT3 inhibitor | AURKA, FLT3, KDR, PDGFRA, SRC, CSF1R, EPHA1, FGFR1, FGFR2, FGFR3, FLT4, KIT, PTK2 | FLT3 inhibitor, VEGFR inhibitor, Aurora kinase inhibitor |
| 7888 | -98.01 | cp | BRD-K11636097 | JNJ-7706621 | CDK inhibitor | CDK1, CDK2, AURKA, AURKB | CDK inhibitor |
| 7468 | -94.41 | cp | BRD-K77286328 | reversine | Aurora kinase inhibitor | AURKB, ADORA3, AURKA, INCENP, MAP2K1 | Aurora kinase inhibitor |
| 6847 | -84.53 | cp | BRD-K24576554 | AT-9283 | JAK inhibitor | AURKA, AURKB, ABL1, BCR, FLT3, JAK2, JAK3, RPS6KA6, STK17A | JAK inhibitor, Aurora kinase inhibitor, ABL inhibitor, BCR-ABL kinase inhibitor, FLT3 inhibitor, Mitotic inhibitor, Protein kinase inhibitor |
| 6787 | -83.29 | cp | BRD-K07881437 | danusertib | Aurora kinase inhibitor | AURKA, AURKB, AURKC, FGFR1, NTRK1, RET, BCR, SLK | Aurora kinase inhibitor, Growth factor receptor inhibitor |
| 6096 | -67.69 | cp | BRD-K36740062 | GSK-1070916 | Aurora kinase inhibitor | AURKB, AURKC, AURKA, CYP2D6, CYP3A4 | Aurora kinase inhibitor |
| 6022 | -66.14 | cp | BRD-K83972459 | JWE-035 | Aurora kinase inhibitor | AURKA | Aurora kinase inhibitor |
| 5760 | -59.65 | cp | BRD-K59184148 | SB-216763 | Glycogen synthase kinase inhibitor | GSK3B, CCNA2, CDK2, GSK3A | Glycogen synthase kinase inhibitor |
